# Clinical and Microbiological Features of Adult Patients With Septic Arthritis of Native Joints at Sultan Qaboos University Hospital: A 10-Year Retrospective Study

**DOI:** 10.7759/cureus.111150

**Published:** 2026-06-19

**Authors:** Rawan Al Busaidi, Turkiya Al-Siyabi, Hashim Ba Wazir

**Affiliations:** 1 General Medicine, Sultan Qaboos University, Muscat, OMN; 2 Microbiology and Immunology, Sultan Qaboos University, Muscat, OMN; 3 Infectious Disease, Sultan Qaboos Hospital, Salalah, OMN

**Keywords:** microbiology, oman, septic arthritis, staphylococcus aureus, synovial fluid culture

## Abstract

Objectives

The study aims to identify the microbiological characteristics, clinical features, and outcomes of adult patients with septic arthritis (SA) at Sultan Qaboos University Hospital (SQUH), Muscat, Oman, over a 10-year period from 2011 to 2020.

Methods

The study is an observational retrospective study that was conducted by retrospectively reviewing culture-positive SA cases treated at SQUH from 2011 to 2020. The data were collected from the hospital medical record (InterSystems TrakCare®; InterSystems Corporation, Cambridge, MA, USA) for patients whose ages were ≥18 years at the time of admission. The study was approved by the Medical Research and Ethics Committee of the College of Medicine and Health Sciences at SQU.

Results

A total of 57 adult patients (41 males, 71.9%) were identified to have native joint SA. The median age was 50 years. Among these patients, 24 patients (42%) had pre-existing joint disease, most commonly osteoarthritis (13 cases, 22.8%). Diabetes (23 patients, 40.4%) and sickle cell disease (SCD) (11 patients, 19.3%) were the most common comorbid conditions. A single joint was involved in 49 cases (86.0%). The most involved joints were the knee (34 cases, 59.6%), the hip (seven cases, 12.3%), and the shoulder (five cases, 8.8%). The most common causative pathogens were *Staphylococcus aureus* in 17 cases (29.8%), followed by *Pseudomonas aeruginosa* in nine cases (15.8%). Most patients underwent surgical interventions, including arthroscopic washout in 39 cases (68.4%) and arthrotomy in five cases (8.8%). The median duration of intravenous antibiotics was 14 days, and the median duration of oral antibiotics was 14 days. Seven cases were treated with antibiotics only without any surgical intervention. The mortality rate was 7%.

Conclusion

In this study, SA was more commonly observed among elderly patients and those with comorbidities, particularly diabetes mellitus and SCD. *S. aureus* was the most common organism.

## Introduction

Septic arthritis (SA) is a serious infection caused by the invasion of a joint by microorganisms, causing inflammation of the synovial membrane. SA has a higher incidence among the elderly and those with comorbid conditions [1,2]. SA can cause cartilage destruction within one to two days of the onset of symptoms and is associated with a considerable mortality rate of up to 11% [3].

SA is associated with several risk factors, including advanced age [4] and preexisting joint diseases such as rheumatoid arthritis (RA) and osteoarthritis [5]. Other significant comorbidities linked to SA include diabetes mellitus, renal failure, and cirrhosis [6]. Furthermore, recent joint surgery is a known risk factor, with an estimated incidence of 14 cases per 10,000 procedures [7].

Accurate diagnosis of SA is based on clinical features such as joint pain, swelling, restricted range of movement, erythema, and increased local temperature. Arthrocentesis and synovial fluid analysis are essential for identifying the causative microorganism [6]. While SA is usually monoarticular, polyarticular involvement can occur, typically in patients with significant comorbidities. The most affected joints are the large joints, such as the knees and hips, followed by the shoulders, wrists, and ankles [8].

The most common causative organism is *Staphylococcus aureus* (65%) [1], followed by *Streptococcus* species (21.95%) [2]. Gram-negative bacilli are a less frequent cause (<20%), with *Proteus mirabilis*, *Klebsiella*, *Escherichia coli*, and *Enterobacter* being the most frequently isolated organisms among them [9].

Prompt targeted therapy is essential for recovery and significantly reduces the risk of complications and mortality. Management typically consists of surgical drainage combined with a prolonged course of antimicrobial therapy [10].

There is a paucity of data regarding the clinical and microbiological features of SA in Oman. Consequently, this study aims to describe clinical presentations, predisposing factors, microbiological profiles, management strategies, and clinical outcomes of adult patients with native joint SA at Sultan Qaboos University Hospital (SQUH) in Muscat, Oman, over a 10-year period.

## Materials and methods

Setting and design

This is a retrospective observational study. It was conducted at SQUH, which is a 700-bed tertiary care teaching hospital in the governorate of Muscat, Sultanate of Oman, over a period of 10 years from 2011 to 2020.

Primary and secondary objectives

The primary objective of this study is to describe the clinical characteristics and risk factors associated with SA in adult patients admitted to SQUH. The secondary objectives of this study are: (i) to identify the pathogens causing SA in adult patients admitted to SQUH, and (ii) to outline the management strategies for SA at SQUH.

Study population

The study included all adult patients ≥ 18 years with confirmed SA by positive synovial culture and suggestive clinical features. Pediatric cases (< 18 years) and prosthetic joint infections were excluded.

Microbiological methods

Synovial fluid samples were processed according to existing laboratory protocols. In brief, the total white blood cell (WBC) counts were performed manually using a Fuchs-Rosenthal counting chamber, and differential counts were conducted following cytospin with Gram staining. The presence of organisms was noted and reported. Samples were cultured on 5% Sheep Blood Agar, Chocolate Agar, Cysteine Lactose Electrolyte Deficient (CLED) Agar, and Sabouraud Dextrose Agar, which were incubated in CO_2_ at 37°C. Additionally, samples were incubated anaerobically at 37°C. When sufficient volume was received, enrichment culture was performed using BD Bactec™ Peds Plus blood culture bottles (Becton, Dickinson and Company, Franklin Lakes, NJ, USA). The samples were incubated for 48 hours prior to 2016; however, this was extended to four days starting in 2016. Organism identification was achieved using phenotypic features, biochemical tests, and an automated identification system (Phoenix™ BD Diagnostics; Becton, Dickinson and Company, Franklin Lakes, NJ, USA). Matrix-assisted Laser Desorption Ionization-Time of Flight (BD™ Bruker MALDI Biotyper® CA System; Becton, Dickinson and Company, Franklin Lakes, NJ, USA) was introduced in 2018 for identification. Susceptibility testing was conducted following the Clinical and Laboratory Standards Institute guidelines (CLSI, M100 and M45).

Data collection

Data were collected from the SQUH hospital information system (InterSystems TrakCare™; InterSystems Corporation, Cambridge, MA, USA), which included demographic data such as age and sex, clinical comorbidities at the time of admission, laboratory features including WBC count, erythrocyte sedimentation rate (ESR), and C-reactive protein (CRP), microbial and synovial fluid data, joint involvement, duration of antibiotic and operative intervention.

Data analysis

Qualitative variables were described using frequencies and percentages. Quantitative variables were described using mean and standard deviation. Frequency tables were used to get the median and the interquartile range (IQR). Analyses were conducted using IBM SPSS Statistics for Windows, Version 28 (Released 2021; IBM Corp., Armonk, NY, USA).

Ethical approval

Ethical approval was obtained from the Medical Research Ethics Committee (MREC) at the College of Medicine and Health Sciences (CoMHS) in September 2021 (MREC#2571).

## Results

Demographic features

A total of 107 patients were identified with SA from 2011 to 2020. Fifty patients were excluded because they did not meet the inclusion criteria; 23 had periprosthetic joint infection, 19 were pediatric cases, four cases did not complete their treatment at SQUH, and four cases were excluded for other reasons. Fifty-seven cases met the inclusion criteria. There were 41 (71.9%) males and 16 (28.1%) females. The median age was 50 years (IQR, 33.5-65.0 years). Twenty-two (38.6%) cases of SA occurred in patients ≥60 years old.

Risk factors and underlying conditions

Pre-existing joint conditions were noted in 24 cases, comprising osteoarthritis in 13 cases (22.8%), avascular necrosis in four cases (7%), trauma in five cases (8.8%), and gout in two cases (3.5%).

Five patients were taking steroids. Five (8.8%) patients developed SA after intra-articular injection. Six (10.5%) cases had a positive history of intravenous drug abuse (IVDU). Regarding systemic diseases, 26 (45.6%) were hypertensive, and 23 (40.4%) had diabetes; 11 (19.3%) cases had sickle cell disease (SCD). A couple of cases had solid cancer, one with a tumor at the head of the pancreas, and the other had breast cancer. Three cases had hematological cancer, including hairy cell leukemia, myelodysplastic syndrome, and multiple myeloma (Table 1). In 17 cases, additional sources of infection were identified, including bedsores, foot ulcers, cholangitis, and cellulitis.

**Table 1:** Medical comorbidities in 57 patients with septic arthritis

Comorbidity	n (%)
Hypertension	26 (45.6)
Diabetes Mellitus	23 (40.4)
Osteoarthritis	13 (22.8)
Sickle Cell Disease	11 (19.3)
Coronary Artery Disease	6 (10.5)
IV Drug Use	6 (10.5)
Intra-articular Injection	5 (8.8)
Trauma	5 (8.8)
Avascular Necrosis	4 (7.0)
Dialysis	3 (5.3)
Hematological Cancer	3 (5.3)
Solid Cancer	2 (3.5)
Gout	2 (3.5)
Osteoporosis	1 (1.8)

Clinical characteristics

**Figure 1:**
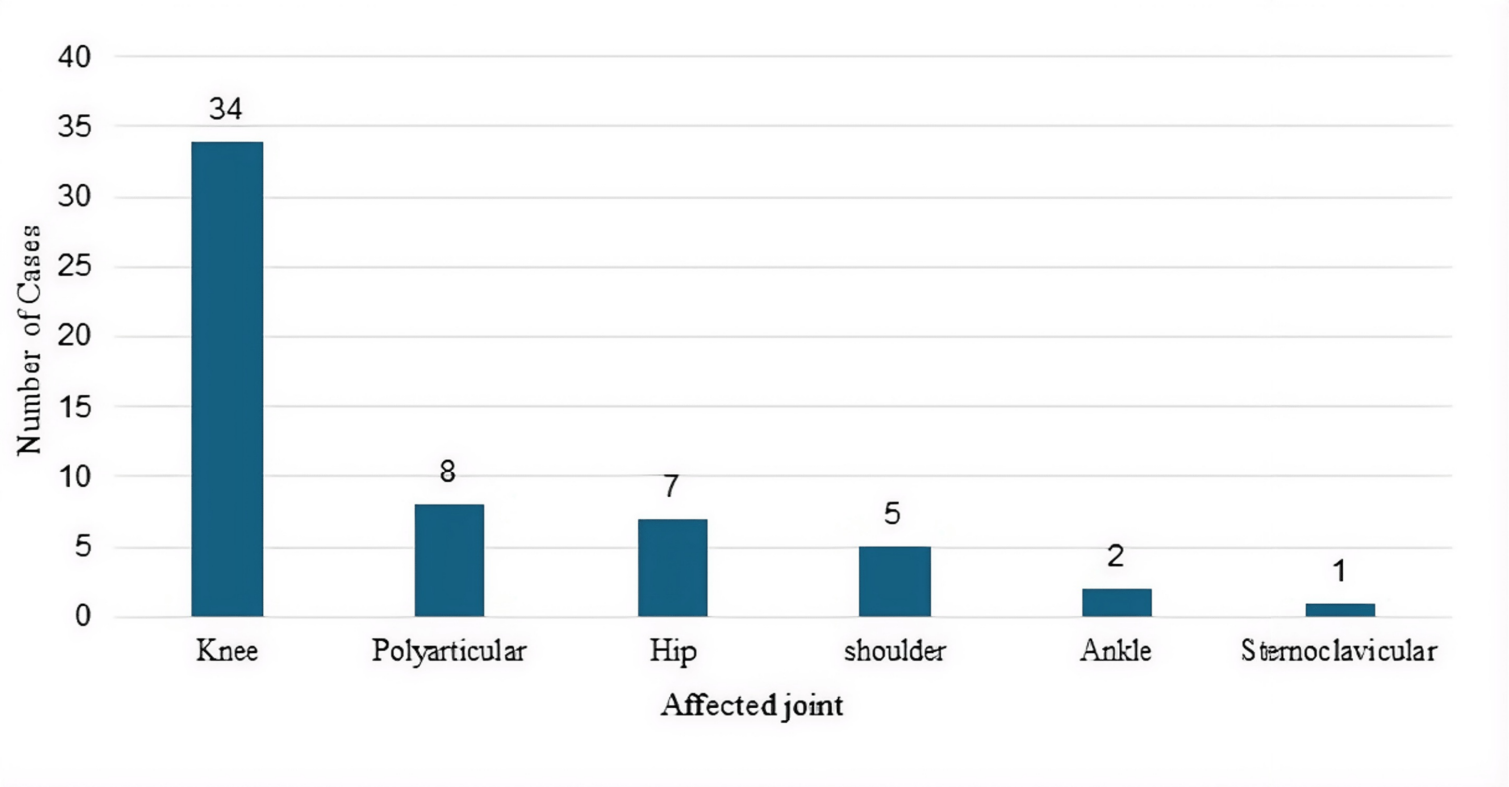
Sites of infection in 57 patients with acute septic arthritis

SA affected single joints in 49 cases (86.0%) and multiple joints in eight cases (14.0%). The most frequently affected joints were the knee in 34 cases (59.6%), the hip in seven cases (12.3%), and the shoulder in five cases (8.8%) (Figure 1). Almost all cases presented with swelling and joint pain.

Microbiological investigation

The predominant pathogens identified were *S. aureus* in 17 cases (29.8%), and *Pseudomonas aeruginosa* in nine cases (15.8%). Methicillin-resistant *S. aureus* (MRSA) was identified in six (10.5%) of cases. *Brucella *species was the causative organism in five cases (8.8%). Only one case was infected with multiple pathogens, namely *Klebsiella pneumoniae* and *Staphylococcus hominis* (Table 2). Fourteen (24.6%) cases had concomitant bacteremia with the same organism isolated from the synovial fluid.

**Table 2:** Distribution of causative organisms of septic arthritis among 57 cases

Pathogens	n (%)
Staphylococcus aureus	17 (29.8)
Pseudomonas aeruginosa	9 (15.8)
*Brucella* species	5 (8.8)
*Achromobacter* species	3 (5.3)
Klebsiella pneumoniae	3 (5.3)
*Salmonella* species	3 (5.3)
Coagulase-negative staphylococci	3 (5.3)
Group B *Streptococcus*	2 (3.5)
Candida parapsilosis	1 (1.8)
Candida tropicalis	1 (1.8)
*Corynebacterium* species	1 (1.8)
Escherichia coli	1 (1.8)
Leclercia adecarboxylata	1 (1.8)
*Acinetobacter* species	1 (1.8)
*Citrobacter* species	1 (1.8)
Pseudomonas putida	1 (1.8)
Streptococcus pneumoniae	1 (1.8)
Streptococcus sanguinis	1 (1.8)

Laboratory investigation

The mean values of laboratory parameters were as follows: CRP, 168 ± 115.65 mg/L; ESR, 85.53 ± 30.37 mm/hr; and leukocyte count, 10.57 × 10^9^/L (Table 3). Synovial fluid WBC count was not available in most cases, as samples were reported to be clotted. Of those analyzable samples, the mean percentage of synovial fluid polymorphonuclear cells (PMNs) was 89.4%.

**Table 3:** Laboratory investigations

Parameter	Value (mean)	Reference range
C-reactive protein	168 ± 115.65 mg/L	0-5 mg/L
Erythrocyte sedimentation rate	85.53 ± 30.37 mm/hr	0-30 mm/hr
WBC count	10.57 × 10^9^/L	2.2-10 × 10^9^/L

Treatment

The median duration of intravenous antibiotics was 14 days (IQR 8-28), and the median duration of oral antibiotics was also 14 days (IQR 14-33). Nineteen cases (33.3%) received IV antibiotics, with no oral antibiotics. Fifty patients (87.7%) were treated with surgery and antibiotics, whereas seven cases were treated only with antibiotics. Arthroscopic washout was done in 39 cases (68.4%) and arthrotomy in five cases (8.8%). One of the cases of polyarticular SA was treated with knee arthroscopic washout and elbow arthrotomy.

Outcome

Eight patients required admission to the intensive care unit (ICU). Four patients died (7%) (Table 4). With regards to mortality cases, three of them occurred in patients aged 70 years and above. They were infected with different organisms as follows: *S. aureus*, *K. pneumoniae*, *Pseudomonas putida*, and MDR *Citrobacter *species. All of them had concomitant bacteremia. The first patient had diabetes, the second patient had diabetes and pancreatic cancer, the third patient had myelodysplastic syndrome, and the last one had SCD. The median length of hospital stay was 19 days (IQR 11.5- 35.5).

**Table 4:** Clinical outcome of septic arthritis in 57 patients

Outcome	n (%)
ICU admission	8 (14)
Death	4 (7)
No follow-up	9 (15.8)

## Discussion

This study identified 57 adult cases with native joint SA over a period of 10 years. The result of this study showed a higher prevalence of SA in males (71.9%); similar studies conducted in the UK [10] and New Zealand [11] found comparable results, with rates of 60% and 66.9%, respectively. The median age in this study was 50 years, closely aligning with the median age of 51.04 years reported in India [6], but exceeding the median age of 44.2 years observed in Saudi Arabia [12].

In the present study, 38.6% of patients were older than 60 years. The increased prevalence of SA cases in the elderly may be explained by increasing comorbidities, degenerative joint diseases, and orthopedic procedures. The most common comorbidities in our study were diabetes mellitus and SCD, and the most common rheumatological disease was osteoarthritis. RA has been identified as an important risk factor in several studies [1,11]. However, in this study, no cases of RA were identified. SCD was an important risk factor for SA in our study. This is likely due to recurrent osteoarticular vaso-occlusive crises [13]. *Salmonella* was the causative pathogen in two cases of SA related to SCD in our study. Nontyphoidal *Salmonella* species are well known to cause osteoarticular infection in patients with SCD [14]. This study also highlighted the association between SA and IVDU; 10.5% of cases were found to be IV drug abusers, similar to what was reported in a British study (15% of cases) [3]. In our study, the most common organism in IVDU-related SA was *P. aeruginosa*. This contrasts with a study by Peterson et al., which found that *S. aureus* was the most common organism causing SA in IVDU [15]. The reasons for this observation remain unclear and cannot be determined from the available data. Potential explanations include local epidemiological factors, differences in injection practices, or environmental exposures; however, these hypotheses were not evaluated in the present study. Furthermore, the relatively small number of intravenous drug users limits the interpretation of this finding. Nevertheless, this observation highlights the potential for regional variation in the microbiology of SA and warrants further investigation in larger studies specifically examining risk factors and pathogen distribution among intravenous drug users.

In our cohort, 8.8% of SA cases were related to intra-articular injections. Iatrogenic SA is a well-documented complication of joint surgeries and injections. A study in Iceland showed that the number of iatrogenic infections increased from 2.8 cases/year in 1990-1994 to 9.0 cases/year in 1998-2002 [7]. This could be explained by the increase in the number of procedures performed each year.

The definitive diagnosis of SA depends on the isolation and identification of the pathogen in synovial fluid. *S. aureus* was found to be the most common infecting organism, in accord with the findings of several other studies [1,3,12]. In our study, 8.8% of patients had *Brucella* SA. Osteoarticular involvement is the commonest form of focal brucellosis, primarily causing spondylitis and sacroiliitis. Peripheral arthritis has been reported in ~39% of cases of osteoarticular brucellosis [16]. One of these cases involved the sternoclavicular joint. Involvement of this joint is uncommon but a well-described complication of brucellosis [17].

At present, there is no evidence from clinical trials to guide the optimal treatment duration of antibiotics [18,19]. As per British Society of Rheumatology guidelines published in 2006, patients should receive IV antibiotics for two weeks followed by oral antibiotics for four weeks [18]. European Bone and Joint Infection Society (EBJIS) 2023 guideline recommends intravenous antibiotics for one to two weeks, and switch to oral treatment as soon as there is clinical improvement, and a treatment duration of oral antibiotics of two to four weeks [19]. The median duration of oral treatment in our study was 14 days, which is at the lower end of EBJIS recommendations and shorter than that suggested by BSR guidelines; however, there was variability in practice, with some patients receiving extended courses based on clinical course and treating physician judgment. This heterogeneity reflects real-world, individualized management rather than a uniform treatment protocol. Although clinical outcomes in our cohort were generally favorable, including a low mortality rate, relapse rates and long-term outcomes were not systematically assessed. Consequently, no definitive conclusions can be drawn regarding the adequacy of shorter treatment durations. These findings should therefore be interpreted as descriptive of current practice patterns rather than as evidence supporting reduced-duration therapy.

Seven cases received antibiotics only, with no surgical intervention. In general, joint drainage is warranted in cases of SA, as this condition is considered a closed abscess. This can be achieved by needle aspiration, arthroscopic drainage, and arthrotomy. A retrospective study by Mabille et al. showed similar efficacy of arthrocentesis vs surgical drainage [20].

This study has several limitations, including its retrospective design and single-center setting. A key limitation is the inclusion of only culture-positive cases of SA. While this approach enhances diagnostic specificity and minimizes misclassification, it may introduce selection bias by excluding clinically suspected culture-negative cases, which can occur due to prior antibiotic exposure, low bacterial burden, or infection with fastidious organisms. Consequently, the true disease burden may be underestimated, and the observed microbiological distribution may be skewed toward more readily cultivable pathogens.

In addition, microbiological techniques evolved over the study period, which may have influenced pathogen detection rates, particularly for fastidious organisms and fungal pathogens, thereby potentially affecting the observed microbiological spectrum. The relatively small sample size may have limited the statistical power and generalizability of the findings; therefore, the results should be interpreted with caution, and further studies involving larger cohorts are warranted to validate these observations.

Another limitation was the high proportion of unavailable synovial fluid WBC counts. Most missing values resulted from synovial fluid specimens being reported as clotted, precluding accurate cell count analysis. This limited the evaluation of an important diagnostic marker for SA and may have reduced the completeness of the assessment of synovial fluid characteristics.

Furthermore, the loss to follow-up rate of 15.8% may have introduced outcome ascertainment bias, as complete follow-up data were unavailable for all patients. Consequently, the reported outcomes should be interpreted with caution. Finally, although this study was conducted at a single tertiary referral center, ensuring a consistent standard of care and diagnostic approach, this may limit the generalizability of the findings to primary or community healthcare settings. Collectively, these factors should be considered when interpreting the results.

## Conclusions

To our knowledge, this study is the first to describe the epidemiology, clinical features, and outcome of SA of native joints in Oman. The most frequently observed comorbidities were diabetes mellitus and SCD. *S. aureus* was the most commonly isolated pathogen, and the knee was the joint most often affected. The majority of patients were managed with arthroscopic washout, followed by two weeks of intravenous antibiotic therapy and an additional two weeks of oral antibiotics.

## References

[1] Dubost JJ, Couderc M, Tatar Z, Tournadre A, Lopez J, Mathieu S, Soubrier M (2014). Three-decade trends in the distribution of organisms causing septic arthritis in native joints: single-center study of 374 cases. Joint Bone Spine.

[2] Muñoz-Egea MC, Blanco A, Fernández-Roblas R (2014). Clinical and microbiological characteristics of patients with septic arthritis: a hospital-based study. J Orthop.

[3] Gupta MN, Sturrock RD, Field M (2001). A prospective 2-year study of 75 patients with adult-onset septic arthritis. Rheumatology (Oxford).

[4] Kaandorp CJ, Van Schaardenburg D, Krijnen P, Habbema JD, van de Laar MA (1995). Risk factors for septic arthritis in patients with joint disease. A prospective study. Arthritis Rheum.

[5] Kaandorp CJ, Krijnen P, Moens HJ, Habbema JD, van Schaardenburg D (1997). The outcome of bacterial arthritis: a prospective community-based study. Arthritis Rheum.

[6] Madi S, Natarajan S, Murali S (2020). A 10 Year clinical, laboratory and arthroscopic data analysis of bacterial septic arthritis of adult native knee: a hospital-based study. J Arthrosc Jt Surg.

[7] Geirsson AJ, Statkevicius S, Víkingsson A (2008). Septic arthritis in Iceland 1990-2002: increasing incidence due to iatrogenic infections. Ann Rheum Dis.

[8] García-Arias M, Balsa A, Mola EM (2011). Septic arthritis. Best Pract Res Clin Rheumatol.

[9] Tarkowski A (2006). Infection and musculoskeletal conditions: infectious arthritis. Best Pract Res Clin Rheumatol.

[10] Rutherford AI, Subesinghe S, Bharucha T, Ibrahim F, Kleymann A, Galloway JB (2016). A population study of the reported incidence of native joint septic arthritis in the United Kingdom between 1998 and 2013. Rheumatology (Oxford).

[11] Kennedy N, Chambers ST, Nolan I, Gallagher K, Werno A, Browne M, Stamp LK (2015). Native joint septic arthritis: epidemiology, clinical features, and microbiological causes in a New Zealand population. J Rheumatol.

[12] Al-Tawfiq JA, Babiker M (2013). Incidence and bacteriologic causes of septic arthritis in a general hospital in Saudi Arabia. Ann Saudi Med.

[13] da Silva Junior GB, Daher Ede F, da Rocha FA (2012). Osteoarticular involvement in sickle cell disease. Rev Bras Hematol Hemoter.

[14] Hernigou P, Daltro G, Flouzat-Lachaniette CH, Roussignol X, Poignard A (2010). Septic arthritis in adults with sickle cell disease often is associated with osteomyelitis or osteonecrosis. Clin Orthop Relat Res.

[15] Peterson TC, Pearson C, Zekaj M, Hudson I, Fakhouri G, Vaidya R (2014). Septic arthritis in intravenous drug abusers: a historical comparison of habits and pathogens. J Emerg Med.

[16] Elzein FE, Sherbeeni N (2016). Brucella septic arthritis: case reports and review of the literature. Case Rep Infect Dis.

[17] Ross JJ, Shamsuddin H (2004). Sternoclavicular septic arthritis: review of 180 cases. Medicine (Baltimore).

[18] Coakley G, Mathews C, Field M (2006). BSR & BHPR, BOA, RCGP and BSAC guidelines for management of the hot swollen joint in adults. Rheumatology (Oxford).

[19] Ravn C, Neyt J, Benito N (2023). Guideline for management of septic arthritis in native joints (SANJO). J Bone Jt Infect.

[20] Mabille C, El Samad Y, Joseph C, Brunschweiler B, Goeb V, Grados F, Lanoix JP (2022). Medical versus surgical treatment in native hip and knee septic arthritis. Infect Dis Now.

